# Striatal Hand Deformity in Parkinson’s Disease Mimicking Rheumatoid Arthritis

**DOI:** 10.31138/mjr.20230804.sh

**Published:** 2023-08-04

**Authors:** Debashis Maikap, Prakashini MV, Prasanta Padhan

**Affiliations:** Department of Clinical Immunology and Rheumatology, Kalinga Institute of Medical Sciences, KIIT University, Bhubaneswar, Odisha, India

**Keywords:** parkinsonism, rheumatoid arthritis, arthritis, deformity

## CLINICAL IMAGE

**Figure 1.**
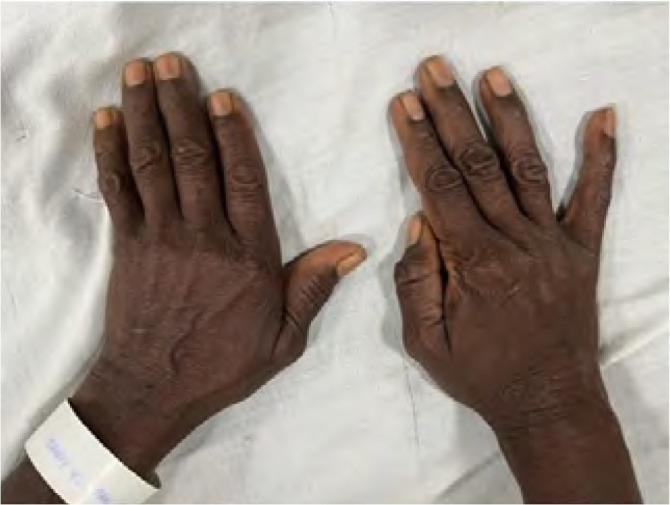
Flexion and ulnar deviation at the MCP joint, correctable Z-deformity of thumb on left hand and flexion of the metacarpophalangeal joint and distal interphalangeal joints and medialisation of thumb (U shaped – “monkey-wrench sign”) at right hand.

A 65-year-old male presented with complaints of painless deformity of right hand followed by left hand for 6 months. He also had pill rolling tremor of right hand with dystonia. He had mask-like face, bradykinesia, cogwheel rigidity seen in both upper limbs, more marked on right side. Secondary causes of parkinsonism such as drugs, trauma, and toxins were excluded. On examination, he had flexion and ulnar deviation at the metacarpophalangeal joint (MCP) joint, correctable Z-deformity of thumb on left hand; flexion of the MCP, and distal interphalangeal joints and medialization of thumb (U shaped – “monkey-wrench sign”) at right hand (**Figure 1**). Investigation revealed normal erythrocyte sedimentation rate, C-reactive protein. Serology for autoimmune diseases such as antinuclear antibody, rheumatoid factor and anti-cyclic citrullinated peptide antibodies were negative. Magnetic resonance imaging of brain was normal. X-ray and musculoskeletal ultrasound of both hands did not show features of inflammatory arthritis.

## DISCUSSION

The term ‘striatal hand’ is originally used by Charcot,^1^ seen in Parkinson’s disease (PD) even in early stage and other atypical parkinsonian syndromes. The term “striatal deformity” suggests that the abnormal postures of the hand, foot, or spine are related to pathology in the part of basal ganglia known as striatum (caudate and putamen) that is frequently associated with various parkinsonian syndromes. The exact pathogenesis is unknown. The pathophysiological origins of striatal deformity appear to be complex and include cortical, and basal ganglia and spinal mechanisms.^2^

Striatal deformities are often misdiagnosed as rheumatoid arthritis, osteoarthritis, Jaccoud’s arthropathy, psoriatic arthritis, Dupuytren’s contracture, and trigger finger because they closely mimic common rheumatologic disorders.^3,4^

In rheumatoid arthritis, the deformities usually involve both hands symmetrically with radiological signs of joint involvement, whereas in striatal hand the deformities are usually unilateral at onset, worse on the side of the PD onset, and on the side with greater PD symptoms and with normal radiological findings.^4^ Levodopa, anti-parkinsonian drug along with joint stretching and range-of-motion exercises, are advised for striatal deformity.^5^ This case highlights that physicians must be conversant with striatal deformities in order to avoid misdiagnosis and order unnecessary investigations and to suggest the most appropriate treatment.
